# Hydrothermal synthesis and characterization of transition metal (Mn/Fe/Cu) co-doped cerium oxide-based nano-additives for potential use in the reduction of exhaust emission from spark ignition engines†

**DOI:** 10.1039/d2ra01954j

**Published:** 2022-05-23

**Authors:** Nazish Qadeer, Naila Jabeen, Latif U. Khan, Manzar Sohail, Muhammad Zaheer, Muhammad Vaqas, Afia Kanwal, Fatima Sajid, Samina Qamar, Zareen Akhter

**Affiliations:** Department of Chemistry, Quaid-i-Azam University (QAU) Islamabad 45320 Pakistan zareenakhter@yahoo.com zareen_a@qau.edu.pk; Nano Sciences and Technology Division, National Centre for Physics QAU Campus, Shahdara Valley Road, P.O. Box 2141 Islamabad 44000 Pakistan naila.jabeen@ncp.edu.pk naila.chem@gmail.com; Synchrotron-Light for Experimental Science and Applications in the Middle East (SESAME) P.O. Box 7 Allan 19252 Jordan; School of Natural Sciences, National University of Sciences and Technology (NUST) H-12 Islamabad Pakistan; SBA School of Science and Engineering, Lahore University of Management Sciences (LUMS) Pakistan; Attock Oil Refinery Limited Morgah Rawalpindi Pakistan

## Abstract

The goal of this work was to synthesize new cerium oxide-based nano-additives to minimise emissions from spark ignition (SI) engines fueled with gasoline blends, such as carbon monoxide (CO), unburned hydrocarbons (HC) and oxides of nitrogen (NO_*x*_). To investigate the effect of transition metal dopants on their respective catalytic oxidation activity, nano-sized CeO_2_ catalysts co-doped with Mn, Fe, Cu and Ag ions were successfully produced by a simple hydrothermal technique. The synthesis of nano-catalysts with cubic fluorite geometry was confirmed by XRD data. The addition of transition metal ions to the CeO_2_ lattice increased the concentration of structural defects like oxygen vacancies and Ce^3+^ ions, which are advantageous for the catalytic oxidation reaction, as also supported by XAFS and RAMAN analysis. Further, nano-gasoline fuel emission parameters are measured and compared to straight gasoline fuel. The results demonstrated that harmful exhaust pollutants such as CO, HC and NO_*x*_ were significantly reduced. The high surface area, better redox characteristics and presence of additional oxygen vacancy sites or Ce^3+^ ions have been linked to the improved catalytic performance of the synthesized catalyst.

## Abstract 2

Illustrating the synthesis of doped and undoped CeO_2_ nanomaterial and its potential application as a promising catalyst for additives to minimize emissions from spark ignition (SI) engines fueled with gasoline blends.

## Graphical abstract

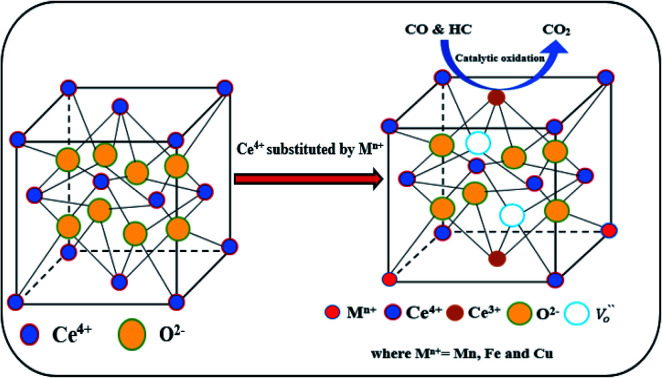

## 1 Introduction

For centuries fossil fuels have played a critical role in the development of power engine applications. However, a gasoline-fuelled spark ignition (SI) engine emits harmful pollutants into the atmosphere such as CO, HC and NO_*x*_ resulting in a variety of worldwide environmental problems such as greenhouse effects, acid rain and foggy weather. As a result, tight emission regulations are implemented to mitigate the effects of these polluting gases. Historically, great efforts have been made to reduce automotive engine emissions of these toxic exhaust gases using three key strategies: (1) internal engine alteration, (2) post-emission treatment of harmful exhaust gases and (3) fuel modification. The current inquiry acquires a gasoline modification strategy since it is simple and does not require extensive internal engine modification.^1^ Recent research has focused on solid nanosized additions as helpful catalysts capable of greatly improving the characteristics of fuel. The addition of a trace amount of nanomaterials to hydrocarbon fuel results in more efficient combustion and lower emissions from automobile engines.^2,3^ The inclusion of nano-additives such as metallic, non-metallic, oxygenates, organic and combinations has been demonstrated in diesel and biodiesel fuel mixes. The acquired results demonstrated an improvement in the physiochemical properties of the fuel, stabilisation of the fuel mixture, and an increase in the heat transfer rate of the fuel. Engine performance parameters also improve as hazardous exhaust emissions are reduced.^4–8^

Due to their low cost, increased redox properties and resistance to sulphur poisoning, metal oxides are the most efficient catalysts as nano-additives in gasoline fuel. Among them, cerium oxide (CeO_2_), a significant rare earth metal oxide, has garnered increased attention in recent decades due to its numerous applications in the field of catalysis, including three-way catalysis (TWC), dehydrogenation reactions, fuel cells, CO oxidation and water gas shift reactions. Cerium oxide's usefulness in pollution reduction and a variety of other sectors is attributed to its unique redox behaviour of oxygen storage and release under oxygen-rich and oxygen-deficient situations, respectively. This distinguishing characteristic is commonly referred to as oxygen storage capacity (OSC). This feature of ceria is related to the generation of oxygen vacancies (*V*_o_) and the low redox potential between Ce^4+^ and Ce^3+^*i.e.*, (1.7 V).^9–13^ However, there are several limitations associated with ceria, in spite of the large number of applications it has. These include a small number of anionic defects, a high working temperature, instability at higher temperatures, and progressive agglomeration of its particles, which results in the eventual deactivation of its redox couple and the subsequent reduction of its optical transparency.^14^

As a result, numerous solutions have been developed to improve ceria's thermal stability and electrical properties. Numerous studies are now being conducted on the doping of CeO_2_ with transition metals. It demonstrates that including less expensive transition elements into the lattice structure of CeO_2_ can enhance ceria's physiochemical properties such as heat stability, redox properties and oxygen vacancies, resulting in increased catalytic activity.^15,16^ Mn, Fe, Co and Cu have received considerable attention due to their changeable oxidation state and better redox behaviour. Additionally, the strong synergetic interactions between the dopant metal oxide and the host CeO_2_ lattice might considerably contribute to increased catalytic activity. As a result of the transition elements' significant oxidative properties combined with oxygen storage materials such as CeO_2_, the resulting mixed oxide acts as an extremely efficient and inexpensive catalyst for oxidation reactions.^17–20^

Recently, Jampaiah *et al.* asserted that the enhanced catalytic performance of copper and manganese doped cerium oxide is due to the production of more Ce^3+^ species or oxygen vacancies (*V*_o_) in the ceria lattice due to the dopant insertion. Furthermore, Yang *et al.* demonstrate that copper doped CeO_2_ has improved catalytic activity due to increased surface oxygen defects in the cerium oxide lattice structure caused by the substitution of Ce^4+^ ions with Cu dopant.^21^ Thus, doping ceria with transition metal cations could result in the formation of Ce^3+^ surface defects or oxygen vacancies (*V*_o_), resulting in a significant increase in the ratio of Ce^3+^/Ce^4+^ species, which is required for the catalytic oxidation activity. Co-doping is the most effective method for increasing the catalytic performance of CeO_2_. Transition metals such as Mn, Fe, Cu have been chosen as possible co-dopants to improve the structural, surface, and redox properties of cerium oxide catalysts.^15–22^ As a result, we synthesized transition metal co-doped CeO_2_ catalysts, such as Mn/Fe–CeO_2_, Mn/Cu–CeO_2_, Fe/Cu–CeO_2_ and Cu/Ag–CeO_2_ by a hydrothermal approach and examined their efficacy in reducing exhaust emissions from spark ignition (SI) engines. The synthesised nano-catalysts were characterised using a variety of techniques, including X-ray diffraction (XRD), RAMAN spectroscopy, X-ray absorption studies (XAFS), X-ray absorption near edge spectroscopy (XANES), extended X-ray absorption fine structure (EXAFS), scanning electron microscopy (SEM), energy dispersive X-ray spectroscopy (EDX), Brunauer–Emmett–Teller (BET) and UV-diffuse reflectance spectroscopy. Further, to the best of our knowledge, for the first time we applied these materials as nano-catalysts in preventing exhaust emission from (SI) engines and found reduced emission of toxic exhaust gases when compared with pure gasoline fuel comprising no catalyst.

## 2 Experimental

### 2.1 Material

A simple hydrothermal method was used to synthesize the transition metals co-doped ceria nano-catalysts. For synthesis, Ce (NO_3_)_3_·6H_2_O, Mn (NO_3_)_4_·4H_2_O, Fe (NO_3_)_3_·9H_2_O, Cu (NO_3_)_2_·H_2_O and AgNO_3_ were employed as precursors of respective metals. All the chemicals purchased from sigma Aldrich and utilized without any process of purification.

### 2.2 Instrumentations and procedures

To obtain powder X-ray diffraction (pXRD) data on manufactured nano-catalysts, we used a source of Cu Kα (0.15418 nm) radiation at 298 K and a PAN analytical X'Pert. Raman measurements were made on Renishaw and the resulting Raman spectra were analysed in the (200–1000) cm^−1^ region utilizing a ViaTM Reflex micro spectrometer. On the beamline of XAFS/XRF, Synchrotron-Light for Experimental Science and Applications in the Middle East, XAFS measurements were made (SESAME). SEM pictures were acquired using a Nova Nanosem 450 field emission scanning electron microscope. Using an accelerating voltage of 10.0 kV, different resolutions were used to capture images from 1 μm, 2 μm, 100 nm and 200 nm. The surface area of samples was determined using a quanta chrome device and the N_2_ adsorption–desorption isotherm (Version 11.04). The optical energy band gap values of nano-catalysts are calculated employing a PerkinElmer Lambda 35 UV-Vis spectrophotometer. Experimental details of XAFS and EXAFS measurements and fit analysis are mentioned in ESI.†

### 2.3 Experimental procedure for exhaust emission analysis

In this analysis, the emission characteristic test has been carried out on a single cylinder four stroke air cooled SI engine with a cylindrical bore of 58 mm and stroke of 56.4 mm, as mentioned in Table 1. The emission parameters have been calculated at a constant speed of 1400 ± 50 rpm for different loads. The engine exhaust was directly discharged into a stainless-steel pipe without any kind of dilution. For each measured emission parameter, the experiment was carried out at least three times. Sampling data for each sample is analysed and collected after the engine has been operated for at least 15 minutes. The whole procedure was carried out in two steps, firstly, preparing the nano-gasoline blends, lastly, measuring the exhaust emissions.

**Table 1:** All the indicators of test engine experiments

Type of engine	Four strokes, air cooled and single cylinder SI engine
Bore	58 mm
Stroke	56.4 mm
Maximal power	11 000 W @ 8500 rpm
Maximal torque	12.76 Nm @ 6500 rpm
Ratio of compression	9.5:1
Fuel injection system	Constant vacuum carburetor
Type of fuel	Gasoline
Idling speed	1400 ± 50
Valve timing	Consumption opens 12.1° crank angle (CA) before top dead center (TDC)
	Consumption closes 55.5° CA after bottom dead center (BDC)
	Exhaust opens 36.5° CA earlier BDC
	Exhaust closes 14.1° CA afterward TDC
Timing of ignition	9.1° CA before TDC

### 2.4 Experimental setup for emission measurement

In the present study, various experimental tests have been performed on SI engines using nano-gasoline fuel blends and pure gasoline to assess the engine emission characteristics. The dosing level of all synthesized nano-additives remains constant *i.e.*, 20 ppm throughout measurements. The SI engine toxic exhaust emissions have been measured by using an exhaust gas analyser of E-Instrument Model 8500. Table 2 gives the specifications of exhaust gas emission analyser. This Model comprises five gas emission analysers which are employed to calculate the emissions of O_2_, HC, CO_2_, CO, and NO_*x*_. For analysing different pollutant gases for instance CO, HC and NO_*x*_, the gas analyser was equipped with online measuring unit cells. The data analysing, processing and calculations were administered by analyser systems to measure oxygen, carbon dioxide and hydrocarbons as percentage volume while carbon monoxide and nitrogen oxide emissions were recorded in ppm.

**Table 2:** Descriptions of exhaust gas analyzer

Sensor	Range (ppm)	Resolution (ppm)	Accuracy (ppm)
Carbon monoxide (CO)	0 to 8000	1.00	<300, (10–8000), 4%
Hydrocarbon (HC)	4000 to 20 000	1.00	>2000, 10%
Nitric oxide (NO)	0 to 4000	1.00	<100, (5–4000), 4%
Nitrogen dioxide (NO_2_)	0 to 1000	1.00	<100, (5–1000), 4%
Hydrogen sulphide(H_2_S)	0 to 500	1.00	<100, (5–500), 4%
Sulphur dioxide (SO_2_)	0 to 4000	1.00	<100, (5–4000), 4%

### 2.5 Nano-catalysts preparation

The catalysts *i.e.*, Mn/Fe–CeO_2_, Cu/Ag–CeO_2_, Fe/Cu–CeO_2_ and Mn/Cu–CeO_2_ were synthesized through facile hydrothermal methods. For the synthesis of Mn/Fe–CeO_2_, Ce (NO_3_)_3_·6H_2_O (5.2 g) was mixed in deionized (DI) water (30 mL). Then the obtained solution stirred for one hour. Next, Mn (NO_3_)_3_·4H_2_O (0.64 g) and Fe (NO_3_)_3_·9H_2_O (1.0 g) were mixed independently in required amount of DI water and transferred simultaneously to the above solution of cerium nitrate and the resulting solution was vigorously stirred for two hours. Furthermore, dropwise addition of aqueous ammonium hydroxide solution to the above solution was carried out at room temperature with continuous stirring until the pH becomes 9. Now, the obtained mixture was later shifted to 100 mL Teflon lined stainless-steel autoclave sealed and thermally heated in a muffle furnace at 200 °C for 24 hours. After heating, the autoclave was cooled down naturally to room temperature. The desired precipitates afterwards separated with ultracentrifugation and washed various times with DI water and later dried in an oven at 100 °C for 12 hours. At last, the dried sample calcined at 500 °C for 5 hours in a furnace to attain the final product *i.e.*, Ce_0.70_Mn_0.15_Fe_0.15_O_2−*δ*_.

Same synthetic procedure was followed for the synthesis of pure CeO_2_, Cu/Ag–CeO_2_, Fe/Cu–CeO_2_ and Mn/Cu–CeO_2_ to obtain the final product *i.e.*, Ce_0.95_Cu_0.04_Ag_0.01_O_2−*δ*_ (A1), Ce_0.85_Fe_0.10_Cu_0.05_O_2−*δ*_ (B2), Ce_0.80_Fe_0.15_Cu_0.05_O_2−*δ*_ (C3) and Ce_0.80_Mn_0.15_Cu_0.05_O_2−*δ*_ (F2) respectively.

## 3 Results and discussion

### 3.1 Characterization studies of nano-catalysts

**Fig. 1:**
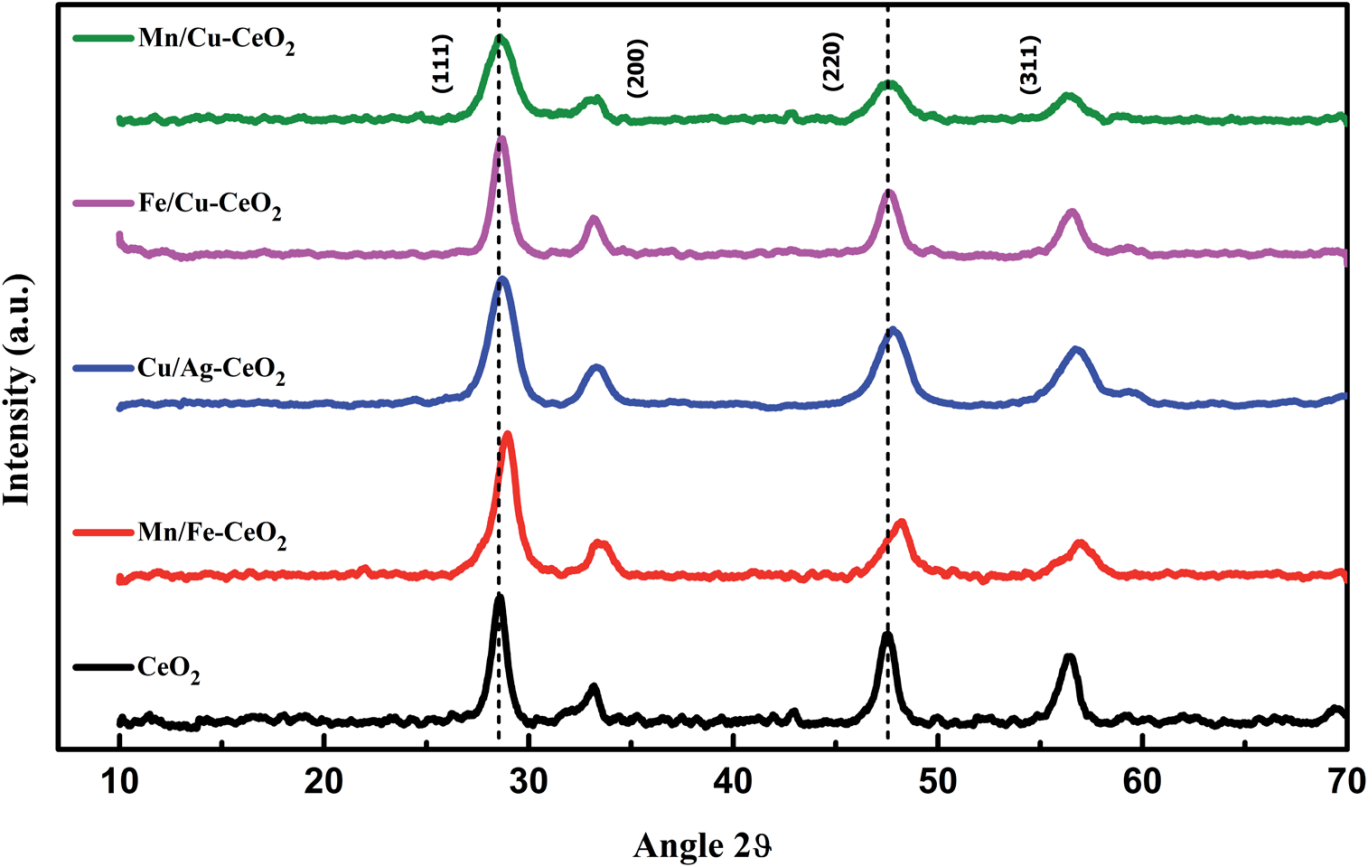
XRD pattern of pure CeO_2_, Mn/Fe–CeO_2,_ Cu/Ag–CeO_2_, Fe/Cu–CeO_2_ and Mn/Cu–CeO_2_.

The crystallinity and geometry of investigated samples have been characterized by XRD. The results of pure CeO_2_ and transition metals co-doped ceria nano-additives are indicated in Fig. 1. XRD pattern of all nano-catalysts revealed intense and sharp peaks at 28.967°, 33.56°, 48.07° and 57.14° which confirm the presence of (111), (200), (220) and (311) planes respectively. These lattice planes are fairly attributed to the cubic fluorite crystal lattice of cerium oxide and each peak is in accordance with the standard reference pattern (JCPDS card number 34-0394). Within the sensitivity of XRD measurement, peaks related to the dopant metal oxide or other impurities are notably absent in the investigated XRD pattern. Fig. 1 also confirms that X-ray diffraction peaks of the samples are slightly shifting towards higher Bragg's angle with reference to pure CeO_2_.

This is due to the shrinkage of ceria lattice structure as the ionic radii of dopants Mn^*x*+^ (Mn^4+^ = 0.53 Å, Mn^3+^ = 0.65 Å, Mn^2+^ = 0.83 Å) and Fe (Fe^2+^ = 0.74 Å; Fe^3+^ = 0.64 Å) are smaller as compared to Ce^4+^ (0.97 Å). Each observation confirms the successful substitution of doped metal oxide into the crystal lattice of ceria to obtain the homogeneous solid solutions of CeO_2_.^11^

Additionally, the XRD pattern of these materials displayed broader peaks than pure ceria, indicating that dopant ions act as a growth and crystallization inhibitor for ceria nanoparticles. To confirm this, the crystallite size of each sample was determined with the highly intense (111) peak of the cubic phase and the well-known Scherrer equation. The average crystallite size of pure CeO_2,_ Mn/Fe–CeO_2_, Cu/Ag–CeO_2_, Fe/Cu–CeO_2_ and Mn/Cu–CeO_2_ was calculated to be 8.98 nm, 8.48 nm, 8.84 nm, 6.19 nm and 5.46 nm respectively, which revealed the nanocrystalline nature of synthesized samples. The smallest particle size of Mn/Cu–CeO_2_ (5.46 nm) than ceria and other transition metal co-doped ceria nano-catalysts suggests the co-doping of Mn/Cu ions assists the dispersion of cerium oxide and notably arrests the growth of ceria crystal, which might be the result of strong synergistic interaction among Ce and Mn/Cu oxide.^11^

**Fig. 2:**
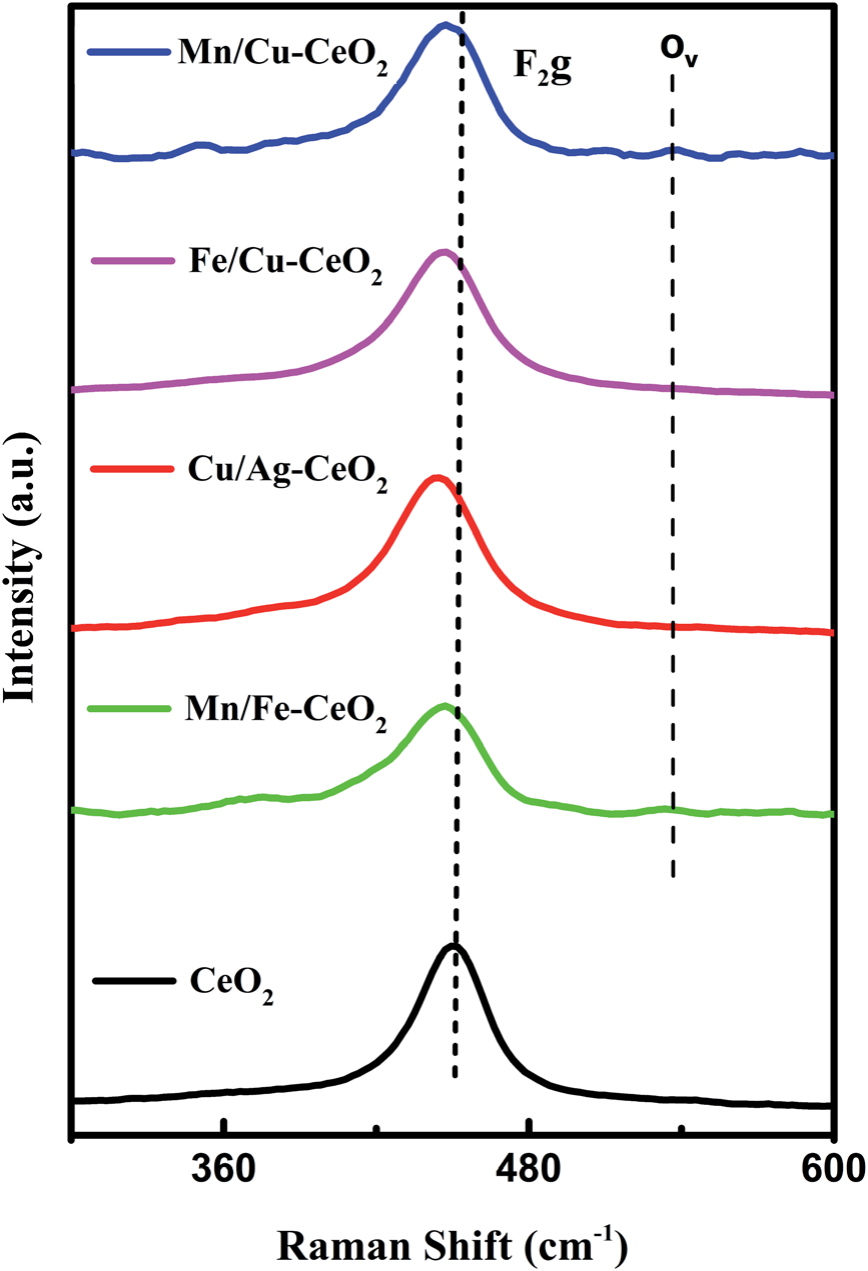
Raman results of undoped CeO_2_, Mn/Fe–CeO_2_, Cu/Ag–CeO_2_, Fe/Cu–CeO_2_ and Mn/Cu–CeO_2_.

Raman analysis is a valuable approach which gives detail associated with the characterization of mixed metal oxides and presence of lattice defects or oxygen vacancies. The Raman spectra of materials are illustrated in Fig. 2. All synthesized materials show a prominent high intense peak for the F_2_g vibrational mode of CeO_2_. In the investigated Raman region, bands corresponding to dopant metal oxides were not identified. This observation confirmed the formation of cerium oxide based solid solutions in line with the XRD results.^11^ However, the shifting of F_2g_ peak position to lower wavenumber (peak position at 443–446 cm^−1^) was observed for transition metal co-doped ceria nano-catalyst as compared to pure CeO_2_*i.e.*, 454 cm^−1^ indicating the defective lattice structure.^18,19^ This redshift can be ascribed to variation of the M–O vibrational frequency, due to ionic radii difference among transition metal dopants and Ce^4+^ ions. The shift also revealed the introduction of Ce^3+^ species or oxygen vacancy sites in the cubic fluorite structure of ceria in order to compensate for the charge imbalance in structure. Moreover, the low intensity peak (O_v_) in case of Mn/Cu–CeO_2_ at 540 cm^−1^ reveals the significant number of oxygen vacancies hence leads to improved catalytic performance for oxidation reaction.^22–25^

### 3.2 Local coordination environment studies of nano-catalysts by XAFS

**Fig. 3:**
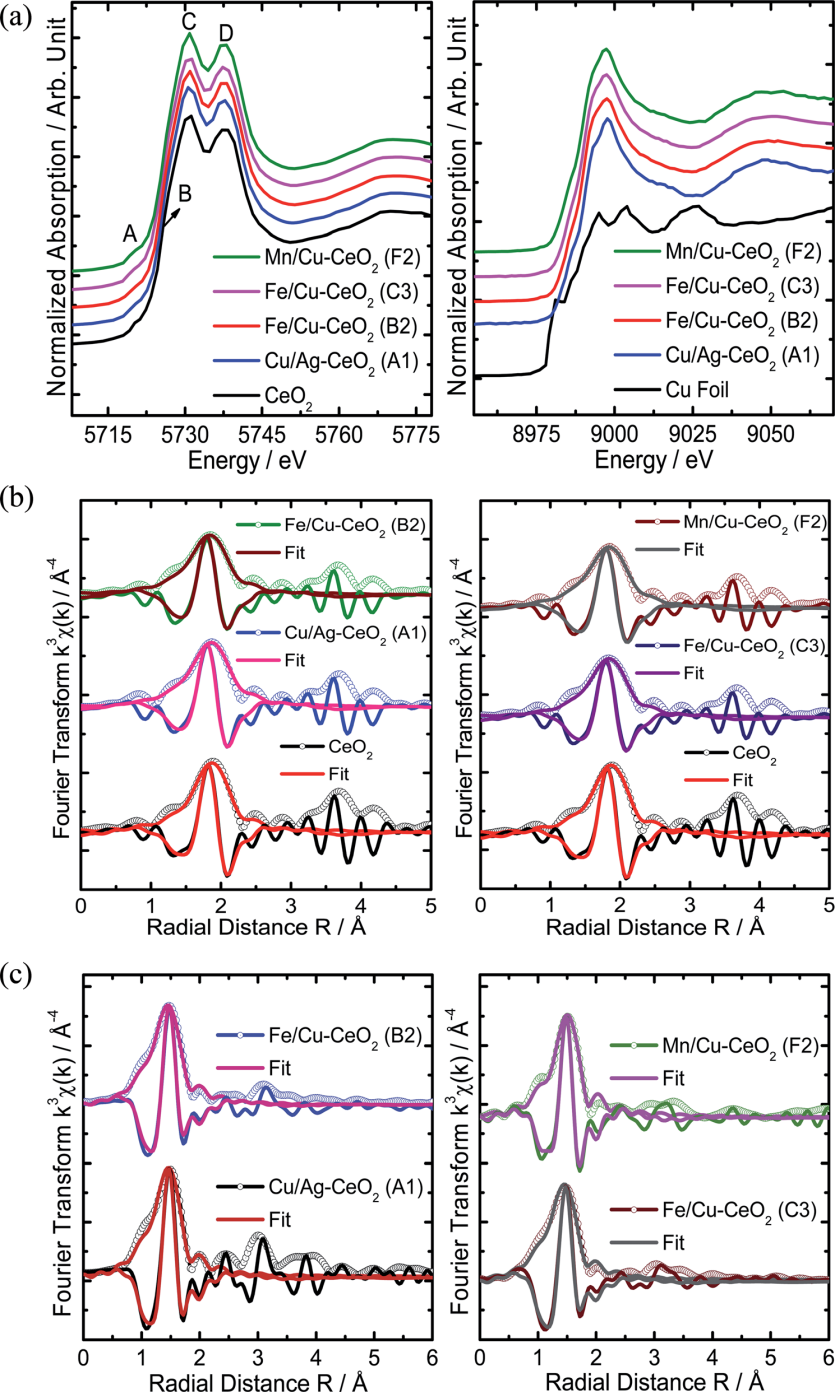
(a) Normalized XANES spectra of the d-transition metal ions co-doped CeO_2_ nanomaterials collected at the Ce L_3_-edge (5723 eV) (left) and Cu K-edge (8979 eV) (right). For clarity the curves are vertically offset. (b) The Fourier transforms of *k*^3^-weighted EXAFS with best fits at Ce L_3_-edge (5723 eV) for the commercial CeO_2_ and d-transition metal ions doped CeO_2_, including (A1) Cu/Ag–CeO_2_, (B2) Fe/Cu–CeO_2_ (left) and (C3) Fe/Cu–CeO_2_, (F2) Mn/Cu–CeO_2_ (right), revealing both the amplitude and the real parts of the Fourier transforms of the data [*χ*(*k*)] and the fits. (c) The Fourier transforms of *k*^3^-weighted EXAFS signals with best fits at the Cu K-edge (8979 eV) for the Cu/Ag–CeO_2_ (A1), Fe/Cu–CeO_2_ (B2) (left) and Fe/Cu–CeO_2_ (C3), Mn/Cu–CeO_2_ (F2) nanomaterials (right), revealing both the amplitude and the real parts of the Fourier transforms of their data [*χ*(*k*)] and the fits.

XAFS spectroscopy is a synchrotron-based method which delivers valuable information about metal definite electronic structure and oxidation state through XANES and variations in the metal local atomic structure represented in EXAFS studies. In Fig. 3(a), the experimental Ce L_3_-edge XANES spectrum of cerium oxide exhibited four distinct A, B, C and D features that are in agreement with the literature reported cubic phase of CeO_2_.^26–28^ The pre-edge peak A is attributed to the 2p_3/2_ → 4f quadrupole transition that arises due to the 5d mixing with the 4f states. Moreover, due to the CeO_2_ cubic lattice, the Ce 5d experiences crystal-field splitting in e_g_ and t_2g_ levels. Therefore, features B and C are assigned to the Ce 2p → 4f^1^5d e_g_-L and 2p → 4f^1^5d t_2g_-L transitions, respectively, whereas L represents a 2p hole of oxygen ligand and 4f^1^ is due to the transfer of an electron from an oxygen 2p orbital to the cerium 4f one. In this case the excitation from the Ce 2p to the 5d is accompanied by an electron excitation/transfer from the O 2p to the Ce 4f orbital, generating a hole in the valence band. The feature D is attributed to the transition from the Ce 2p to the 4f^0^5d state (with no f electrons). Nevertheless, the XANES spectra of d-transition metal ions (Cu, Fe and Mn) co-doped CeO_2_ nanomaterials manifested similar four A, B, C and D features, suggesting the same CeO_2_ cubic structure for these materials.

Alternatively, the Cu K-edge (8979 eV) XANES spectra of all the d-transition metals ions (Cu, Fe and Mn) co-doped CeO_2_ nano-catalysts clearly illustrated the existence of prevailing Cu(ii), resulting in similar broad low energy shoulder in the region below 8985 eV and intense rising edge from 8992 to 9002 eV (Fig. 3(a) right), characteristic of the Cu(ii). The Fe K-edge (7112 eV) XANES spectra of the Fe/Cu–CeO_2_ (B2 and C3) nano-catalysts displayed the weak 1s → 3d pre-edge peaks at ∼7114 eV (ref. 29) and rising edge peaks above 7128 eV (ESI Fig. S1†), manifesting the existence of Fe(iii) in octahedral (O_h_) environment. Whereas, the Mn K-edge (6539 eV) XANES spectrum showed the presence of Mn^2/3+^ in the Mn/Cu–CeO_2_ (F2) nano-catalyst (ESI Fig. S1†). It confirms the variable valence states of Mn(ii and iii) in Mn/Cu–CeO_2_ sample and manifests the existence of Fe(iii) and Cu(ii) ions in Fe/Cu–CeO_2_ and Cu/Ag–CeO_2_ samples respectively. This provides an insight into the existence of facile redox behavior among Mn^3+^/Mn^2+^ ions associated with the redox couple of Ce^4+^/Ce^3+^ in ceria lattice hence leads to the production of increased oxygen vacancies in Mn/Cu–CeO_2_ catalyst, also validated by Raman spectroscopy.^10^ The improved oxygen vacancies in the sample might be catalytically beneficial for minimizing toxic exhaust emission.

To investigate the local chemical environment around Ce ions, the Ce L_3_-edge EXAFS data of all the materials were quantitatively analysed and nonlinear best fit was performed to their Fourier transformed *k*^3^-weighted experimental EXAFS signals, using Artemis program from the Demeter.^30–32^ Fourier transforms of *χ*(*k*) and their corresponding real components with best curve fitting result exhibited the eight backscattering oxygen atoms in the first coordination shell, suggesting CeO_2_ cubic fluorite structure with (225) space group for all the materials^33^ as appear in the below Fig. 3(b). The spectral fits manifested that the bond distances of first coordination shell for the (A1) Cu/Ag–CeO_2_, (B2) Fe/Cu–CeO_2_, (C3) Fe/Cu–CeO_2_ and (F2) Mn/Cu–CeO_2_ were slightly less than the reference CeO_2_, which might be due to slight distortion in the host CeO_2_ lattice due to insertion of d-transition metal ions (Cu, Fe and Mn) dopants (Table 3). In addition, the structural disorder around the Ce is also increased in the Cu/Ag–CeO_2_ (A1), Fe/Cu–CeO_2_ (B2), Fe/Cu–CeO_2_ (C3) and Mn/Cu–CeO_2_ (F2) when compared to the reference CeO_2_, as demonstrated by their higher *σ*^2^ (Debye–Waller factors) values, suggesting defective local atomic structures for all these nano-catalysts. Nevertheless, the overall Ce L_3_-edge XAFS results suggest the existence of Ce sites between Ce^3+^ (4f^1^L) and Ce^4+^ (4f^0^) character, with a hole (L) in the O 2p valence band.^26,27^ Therefore, it validates the co-existence of Ce^4+^ and Ce^3+^ species on the surface of all cerium oxide-based nano-additives. Previous studies specify the existence of reduced cerium cations (Ce^3+^) to the production of oxygen vacancies in ceria lattice which play a key role in catalytic oxidation reaction.^11^

**Table 3:** Derived fitting parameters of EXAFS, involving *N*: coordination number, *R*: mean coordination shell radius, *σ*^2^: mean square relative displacement (MSRD) or Debye–Waller factor, *S*_o_^2^: amplitude reduction factor, *E*_o_: photoelectron energy and *R*_factor_: goodness of the fit for the Ce L3-edge (5723 eV) *χ*(*k*) of CeO_2_ and d-transition metal ions (Cu, Fe and Mn) co-doped CeO_2_

Materials	Bond type	*N*	*R* (Å)	*σ* ^2^ (Å^2^)	*S*_o_^2^	*E*_o_ (eV)	*R*_factor_
CeO_2_	Ce–O	8	2.329 ± 0.009	0.0069 ± 0.0035	0.82	6.69 ± 1.13	0.019
Cu/Ag–CeO_2_ (A1)	Ce−O	6.8	2.320 ± 0.006	0.0080 ± 0.0012	0.86	6.92 ± 1.07	0.017
Fe/Cu–CeO_2_ (C3)	Ce−O	7.5	2.308 ± 0.008	0.0099 ± 0.0014	0.82	5.89 ± 1.48	0.015
Fe/Cu–CeO_2_ (B2)	Ce−O	7.4	2.309 ± 0.007	0.0103 ± 0.0012	0.88	5.91 ± 1.25	0.017
Mn/Cu–CeO_2_ (F2)	Ce−O	7.6	2.317 ± 0.008	0.0113 ± 0.0019	0.89	6.15 ± 1.08	0.019

In an effort to gain understanding on the local environment of Cu, Fe and Mn ions dopants in the CeO_2_ host lattice, the XAFS analyses at the Cu K-edge (8979 eV), Fe K-edge (7112 eV) and Mn K-edge (6539 eV) respectively were employed for all the materials, as indicated in Fig. 3(c) and (ESI Fig. S1†). The Cu K-edge Fourier transforms of *χ*(*k*) of these nano-catalysts clearly demonstrated a dominant peak at ≈1.92 Å, attributed to the Cu–Obond pair, suggesting existence of Cu^2+^ incorporated in the CeO_2_ cubic host lattice. Whereas, a small Cu–Ce contribution from Cu–[O_*x*_]–Ce structures (*R* ≈ 3.3 Å), were also demonstrated, validating the existence of an interaction among copper and ceria, consistent with the previous literature reported EXAFS of copper-ceria.^34^ So as to discover further the effect of Cu dopant ion incorporation on the local electronic structure of CeO_2_, the best fit to the Cu K-edge *χ*(*k*) was carried out in *R*-space from 1.0 to 2.0 Å interval with Hanning window and in the 2–11 Å^−1^*k* range. The similar Cu–O cubic structure of lattice parameters (*a*, *b* & *c* = 4.245 Å) and same *Fm*3̄*m* (225) space group was used to calculate the theoretical values of effective scattering amplitude *F*_i_(*k*), effective scattering phase shift *ϕ*_i_(*k*) and mean free path of the photoelectron *λ via* FEFF8-lite code in Artemis, generating various scattering paths. The first coordination shell of the Cu–O single scattering path was comprised in the best fit analysis, refining the amplitude reduction factor (*S*_o_^2^), energy shift parameter (Δ*E*_0_) and disorder in the bond length (*σ*^2^). Thus, an optimum fit was obtained for all the nano-catalysts, demonstrating that the Cu dopant ion occupies an octahedral symmetry site in the CeO_2_ cubic lattice. However, the shortened Cu–O interatomic distance (∼1.92 Å) when compared to the Ce–O (∼2.3 Å) one in cubic lattice is owning to the lower ionic radius of the Cu^2+^ (0.73 Å) dopant then the Ce^4+^ (0.97 Å) host ion. The lower values of coordination number (<6) suggested the occurrence of oxygen vacancy in first coordination shell near the octahedral (O_h_) Ce site substituted by Cu ion (Table 4). It is well known that oxygen vacancies are beneficial for the activation of surface oxygen species that directly participate in CO and HC oxidation reactions. Additionally, the Fe K-edge and Mn K-edge Fourier transformed EXAFS data of Fe/Cu–CeO_2_ (B2), Fe/Cu–CeO_2_ (C3) and Mn/Cu–CeO_2_ (F2) also exhibited their first dominant peaks from the respective Fe–O and Mn–O bond pairs which are remarkably overlapped with the corresponding Cu–O peak (ESI Fig. S1†), suggesting the same chemical environment around Fe and Mn ions as Cu for all the nano-catalyst.

**Table 4:** Fitting parameters of EXAFS for the Cu K-edge (8979 eV) *χ*(*k*) of d-transition metal ions (Cu, Fe and Mn) co-doped CeO_2_ nanomaterials

Materials	Bond type	*N*	*R* (Å)	*σ* ^2^ (Å^2^)	*S*_o_^2^	*E*_o_ (eV)	*R*_factor_
Cu/Ag–CeO_2_ (A1)	Cu−O	4.5	1.920 ± 0.007	0.0035 ± 0.0023	0.70	0.69	0.020
Fe/Cu–CeO_2_ (B2)	Cu−O	4.0	1.922 ± 0.007	0.0044 ± 0.0022	0.79	0.05	0.010
Fe/Cu–CeO_2_ (C3)	Cu−O	4.0	1.919 ± 0.008	0.0043 ± 0.0025	0.76	0.30	0.015
Mn/Cu–CeO_2_ (F2)	Cu−O	4.0	1.939 ± 0.006	0.0020 ± 0.0002	0.63	2.46	0.012

SEM along with EDX was performed so as to get comprehensive information related to the changes in morphology of samples and elemental chemical compositions of the prepared transition metals co-doped ceria nano-catalysts respectively. The SEM images of investigated nano-additives in Fig. S2 (ESI†) shows nonuniform distribution of clusters of irregularly shaped particles with pronounced agglomeration. Additionally, the EDX pattern of all samples confirms the substitution of Mn, Fe and Cu ions in the crystal lattice of ceria.^22^ Furthermore, Fig. S3 (ESI†) also shows the atomic weight percent of elements Mn, Fe, Cu, Ce and O present in each material according to stoichiometry.

BET surface area analysis of cerium oxide-based nano-additives has been calculated by using nitrogen gas adsorption isotherm. Nitrogen adsorption–desorption isotherm of each sample shows the type IV hysteresis loop which confirms the mesoporous nature of the materials as illustrated in Fig. S4 (ESI†). The surface area of investigated samples has been measured and results are given in Table 5.

**Table 5:** Calculated surface area values of investigated nano-catalysts

Catalysts	Surface area (m^2^ g^−1^)
CeO_2_	41.00
Mn/Fe–CeO_2_	78.093
Fe/Cu–CeO_2_	85.725
Mn/Cu–CeO_2_	91.773
Cu/Ag–CeO_2_	49.514

The above data illustrates that the transition metal co-doping strategy of CeO_2_ nano-catalyst clearly improves the exposed surface area of the catalysts. In general, smaller size particles provide a large surface area catalyst as compared to large size particles. Therefore, Mn/Cu–CeO_2_ has highest surface area (91.733 m^2^ g^−1^) indicating the introduction of Mn and Cu ions into CeO_2_ lattice structure notably resists the crystallite growth, resulting in an enhancement in the surface area of nano-catalyst which is according to the XRD results.^18^ Thus, the catalyst with large surface area facilitates more surface-active sites to the reactant molecule and hence promotes the reaction more beneficially over its surface hence might be the best catalyst for exhaust emission reduction.^35^

**Fig. 4:**
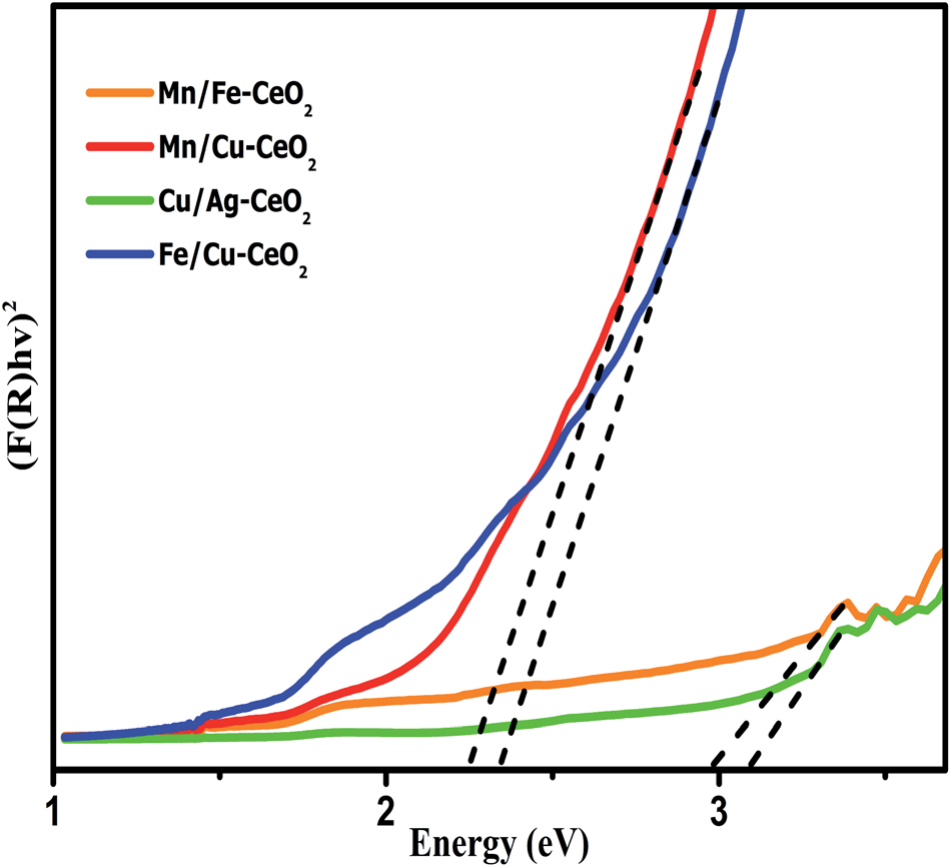
.Band gap energy of transition metals co-doped cerium oxide nano-catalysts.

The effect of incorporation of multivalent transition metal cations into ceria lattice further investigated by using UV-DRS analysis which is achieved in the range of 200–1200 nm. Fig. 4 indicates the UV-DRS spectra of the synthesized samples. The Kubelka–Munk function equation has been utilized to calculate optical band gap (*E*_g_) values of all the nano-catalysts. The observed calculated energy band gap (*E*_g_) values are 2.98 eV, 3.09 eV, 2.35 eV and 2.26 eV for Mn/Fe–CeO_2_, Cu/Ag–CeO_2_, Fe/Cu–CeO_2_ and Mn/Cu–CeO_2_ respectively. The *E*_g_ values of investigated samples are much lower as compared to literature reported values for pure ceria *i.e.*, 3.25 eV. Thus, co-doping strategy results in noticeable reduction in the band gap of ceria. This observed red shift manifested that 3d transition metals provide lower unoccupied orbital than Ce 4f, hence electronic transitions from O 2p to this lower unoccupied orbital needs less energy. Moreover, doping of ceria with Mn, Fe and Cu ions generate oxygen vacancies (*V*_o_) and assist the conversion between Ce^3+^/Ce^4+^ ions.^22^ This enhances the ratio of Ce^3+^ species that ultimately give rise to the production of localized energy states which are nearer towards conduction bands and thus lowering the energy band gap (*E*_g_). These outcomes reveal that the substitution of transition metal co-dopants into ceria matrix significantly improves the crystal structure and electronic characteristics of the CeO_2_.^36,37^

### 3.3 Exhaust emission analysis

**Fig. 5:**
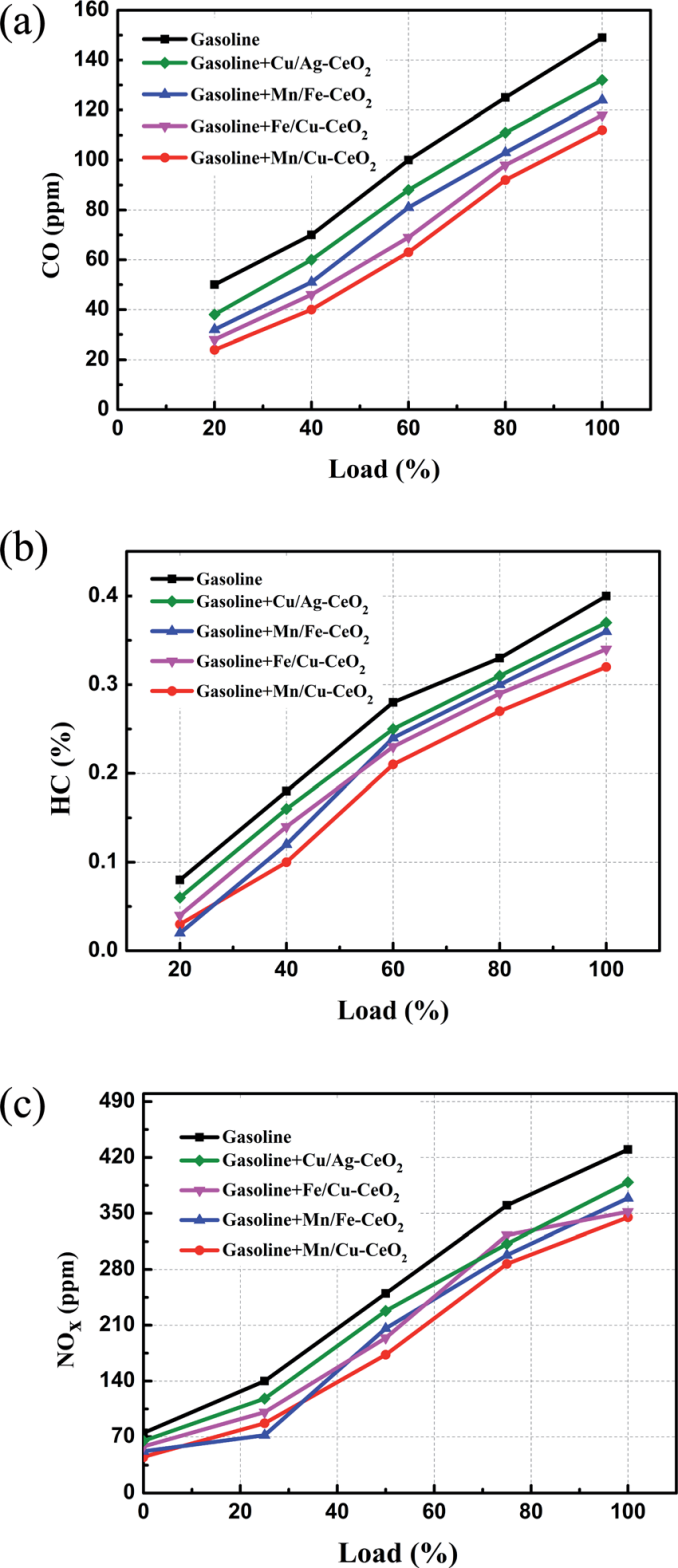
(a) Variations in emission levels of CO with respect to load. (b) Variations in emission levels of HC with respect to load. (c) Variation in the emission level of NO*_x_* with respect to load.

Carbon monoxide is a harmful pollutant gas and its emission must be minimized. It is formed as an intermediate species in the combustion reaction of hydrocarbon fuel therefore, its emission produced from incomplete combustion reaction. The CO emissions from automobiles mainly depend on the equivalence ratio. In fuel-lean mixtures there is an excess amount of oxygen available hence, CO oxidizes and forms carbon dioxide (CO_2_). In fuel rich mixtures, there is deficiency in the availability of oxygen, to fully combust the mixtures of fuel-air. As the concentration of oxygen is low to form CO_2_, the CO emission is enhanced. Fig. 5(a) shows the CO emissions for pure gasoline and nano-gasoline fuel blends. The graph clearly specifies the decrease in CO emissions to some extent compared to neat gasoline. Due to excellent oxygen storage and release capacity of ceria, nano-CeO_2_ based additives act as an oxygen buffer in the gasoline fuel and supply excess oxygen for carbon and leads to the reduction of CO emissions, as revealed in eqn (1).^39^1
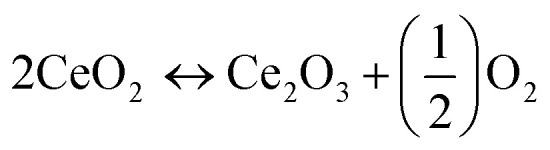


The oxygen vacancy defects of nano-ceria absorbed molecules of carbon monoxide (CO) to directly produce CO_2_ or form the intermediate bidentate carbonate species, which oxidizes and may leads to the development of CO_2_ as illustrated in eqn (2),

2 2CeO_2_ + CO → Ce_2_O_3_ + CO_2_

Fig. 5(b) illustrates the variations in hydrocarbon emissions with respect to load. Hydrocarbon (HC) emission from nano-gasoline fuel blends is much lower as compared to pure gasoline. Unburned hydrocarbon forms because of lack of sufficient oxygen content in combustion of fuel and added nano-additives provide excess oxygen to gasoline so as a result efficient combustion is achieved which ultimately reduces hydrocarbon emission, as mentioned in the following eqn (3).^12^3
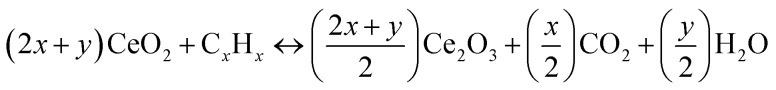


NO_*x*_ emission varies with reference to load as indicated in Fig. 5(c). It is understood from the graph that a lowering in NO_*x*_ emission was evident after the addition of nano-additive in gasoline as compared to base fuel. Owing to high thermal stability of ceria, cerous oxide (Ce_2_O_3_) produced from the oxidation reaction of CO and HC still remains active after increasing the initial combustion reaction and re-oxidized back to CeO_2_*via* the decline in the NO_*x*_ emission level as described in eqn (4). Moreover, this decrease in NO_*x*_ emissions can also be attributed to several other different reasons such as reduction in combustion temperature, peak pressure, reaction time and oxygen content.^38,39^4
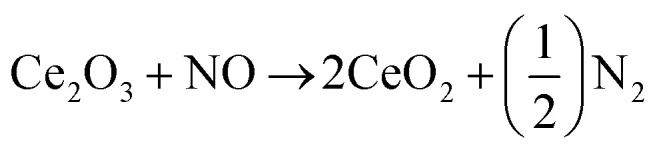


### 3.4 Catalytic activity studies

#### 3.4.1 Emission characteristics

We have evaluated the catalytic efficiency of transition metal co-doped ceria nano-additives for exhasut emission reduction (CO, HC & NO_*x*_) in gasoline fuel. For this purpose, various experimental tests have been carried out on SI engines using gasoline fuel blends and pure gasoline to assess engine emission parameters. The exhaust gas analyser (E-Instrumental Model 8500) processes, calculates and records the data of engine exhaust in order to determine the ppm or percentage of examined gases in each sample. The obtained experimental data is demonstrated as exhaust emission profiles for CO, HC and NO_*x*_ in Fig. 5(a)–(c) to investigate the catalytic performance of cerium oxide-based nano-additives.

In general, the oxidation of CO and HC occurs through the Mars–van Krevelen mechanism,^11^ in which the lattice oxygen of ceria catalysts participates in the chemical reaction and is later supplied by molecular gas phase oxygen as indicated in eqn (1). It demonstrates that the mobility of lattice oxygen has a vital role in influencing the catalytic oxidation activity. Previous research indicates that by generating homogeneous solid solutions with transition metal dopants, the catalytic performance of CeO_2_ can be enhanced. This is accomplished by the strong synergistic interactions between the host CeO_2_ and the dopant metal oxide, which improve the lattice oxygen mobility by enhancing the concentration of oxygen vacancies (*V*_o_) in the synthesized sample.^12^

In co-doped ceria samples, strong synergistic interactions between Ce and d-transition metal ion dopants (Mn, Fe and Cu) enhance Ce^4+^/Ce^3+^ redox activity, which is critical for reducing high-valent cerium ions and releasing oxygen for oxidation reactions. As a result, the improved oxygen buffer capacity of CeO_2_ based nano-additives in gasoline fuel permits simultaneous oxidation of CO and HC (eqn (2) and (3)) as well as a reduction in NO_*x*_ emissions (eqn (4)), decreasing harmful exhaust gases, particularly in the stoichiometric ratio. It is obvious from Fig. 5(a)–(c) that Mn/Cu–CeO_2_ nano-additive exhibits superior catalytic performance compared to other co-doped ceria nano-additives. As explained above from XANES analysis, oxygen vacancies in Mn/Cu–CeO_2_ sample not only formed due to the conversion of Ce^4+^ to Ce^3+^ but also the incorporation of Mn^3+^/Mn^2+^ ions into lattice structure of ceria. As a result, the mobility and amount of oxygen vacancies on the surface of the ceria increases that plays a key role in the catalytic oxidation reaction. Thus, Mn/Cu–CeO_2_ nano-additive is catalytically more active in gasoline fuel as compared to other nano-catalysts and considerably lowers the emissions of CO, HC and NO_*x*_. The higher catalytic activity of the nano-catalyst can be ascribed to the following factor, (1) the significant amount of surface oxygen vacancies which is evident from XAFS, XANES, EXAFS, Raman and UV-DRS spectroscopy (2) the increased specific surface area of prepared samples combined with smaller crystallite sizes contributes to a greater number of surface-active sites, which play a vital role in improving the catalytic activity of the samples.

## 4 Conclusion

To summarize, Mn/Fe, Mn/Cu, Fe/Cu and Cu/Ag co-doped CeO_2_ based nano-additives were successfully produced by facile hydrothermal method and investigated for exhaust emission reduction. XRD results confirm the formation of nanocrystalline solid solutions of ceria with cubic fluorite geometry. UV-DRS analysis demonstrates that chemical co-doping dramatically reduces the band gap of CeO_2_, hence improving its redox characteristics. According to XAFS and Raman analysis, co-doping of multivalent transition metal cations results in the formation of enhanced oxygen vacancies or Ce^3+^ ions on the surface of doped CeO_2_, hence increasing the rate of oxygen transport for oxidation. All of the nano-additives based on cerium oxide were subjected to exhaust emission monitoring in a SI engine. The obtained results reveal that Mn/Cu–CeO_2_ nano-additive exhibits superior catalytic performance and significantly reduces the harmful emission of CO, HC and NO_*x*_ in comparison to pure gasoline. It can be attributed to smaller particle size, higher surface area, better redox properties and large number of oxygen vacancies or Ce^3+^ ions evident from Raman, XAFS, XANES, EXAFS and UV-DRS spectroscopy. Finally, these findings indicate that co-doping may be an effective strategy for developing highly active cerium oxide-based nano-additives capable of achieving complete combustion and so reducing exhaust emissions from SI engines.

## Author contributions

Nazish Qadeer: methodology, formal analysis, writing – the original draft, review & editing. Naila Jabeen: conceptualization, supervision, methodology, resources, validation, writing the draft, review & editing. Latif U. Khan: data curation, analysis, aoftware & writing. Manzar Sohail: data curation. Muhammad Zaheer: data curation & analysis. Muhammad Vaqas: data curation & analysis. Afia Kanwal, Fatima Sajid & Samina Qamar: data analysis. Zareen Akhter: conceptualization, resources, project administration, supervision, writing – review & editing.

## Conflicts of interest

The authors of this manuscript declare that they have no known personal relationships or competing financial interests which can have appeared to influence the work reported in this paper.

## Supplementary Material

RA-012-D2RA01954J-s001
