# Supplementary material for: Hydrothermal synthesis and characterization of transition metal (Mn/Fe/Cu) co-doped cerium oxide-based nano-additives for potential use in the reduction of exhaust emission from spark ignition engines
Source: RSC Adv. 2022 May 23;12(24):15564–74. doi: 10.1039/d2ra01954j (PMC9125985; doi:10.1039/d2ra01954j)
Supplement: RA-012-D2RA01954J-s001 [file RA-012-D2RA01954J-s001.pdf]

Electronic Supplementary Material (ESI) for RSC Advances.
This journal is © The Royal Society of Chemistry 2022

Supporting Information for

Hydrothermal synthesis and characterization of transition metals (Mn/Fe/Cu) co-doped cerium oxide-based nano-additives for their potential use in the reduction of exhaust emission from spark ignition engine

Nazish Qadeer[a], Naila Jabeen[b]*, Latif U. Khan[c], Manzar Sohail[d], Muhammad Zaheer[e], Muhammad Vaqas[f], Afia Kanwal[a], Fatima Sajid[a] Samina Qamar[a] and Zareen Akhter[a]*

Authors Affiliation

[a] Department of chemistry, Quaid-i-Azam university (QAU) Islamabad 45320, Pakistan.

[b] Nanosciences and Technology Division, National Centre for Physics, QAU Campus, Shahdra Valley Road, P.O. Box 2141, Islamabad-44000, Pakistan.

[c] Synchrotron- light for Experimental Science and Applications in the Middle East (SESAME) P.O. Box 7, Allan 19252, Jordan.

[d] School of Natural Sciences, National University of Sciences and Technology (NUST), H-12, Islamabad, Pakistan

[e]SBA School of Science and Engineering, Lahore University of Management Sciences (LUMS).

[f]Attock Oil Refinery Limited, Morgah, Rawalpindi, Pakistan.

Corresponding Authors

Zareen Akhter - Department of Chemistry, Quaid-i-Azam University Islamabad 45320, Pakistan. Email: zareenakhter@yahoo.com & zareen_a@qau.edu.pk

Naila Jabeen - Nanosciences and Technology Division, National Centre for Physics, QAU Campus, Shahdra Valley Road, P.O. Box 2141, Islamabad-44000, Pakistan.

Email: naila.chem@gmail.com & naila.jabeen@ncp.edu.pk

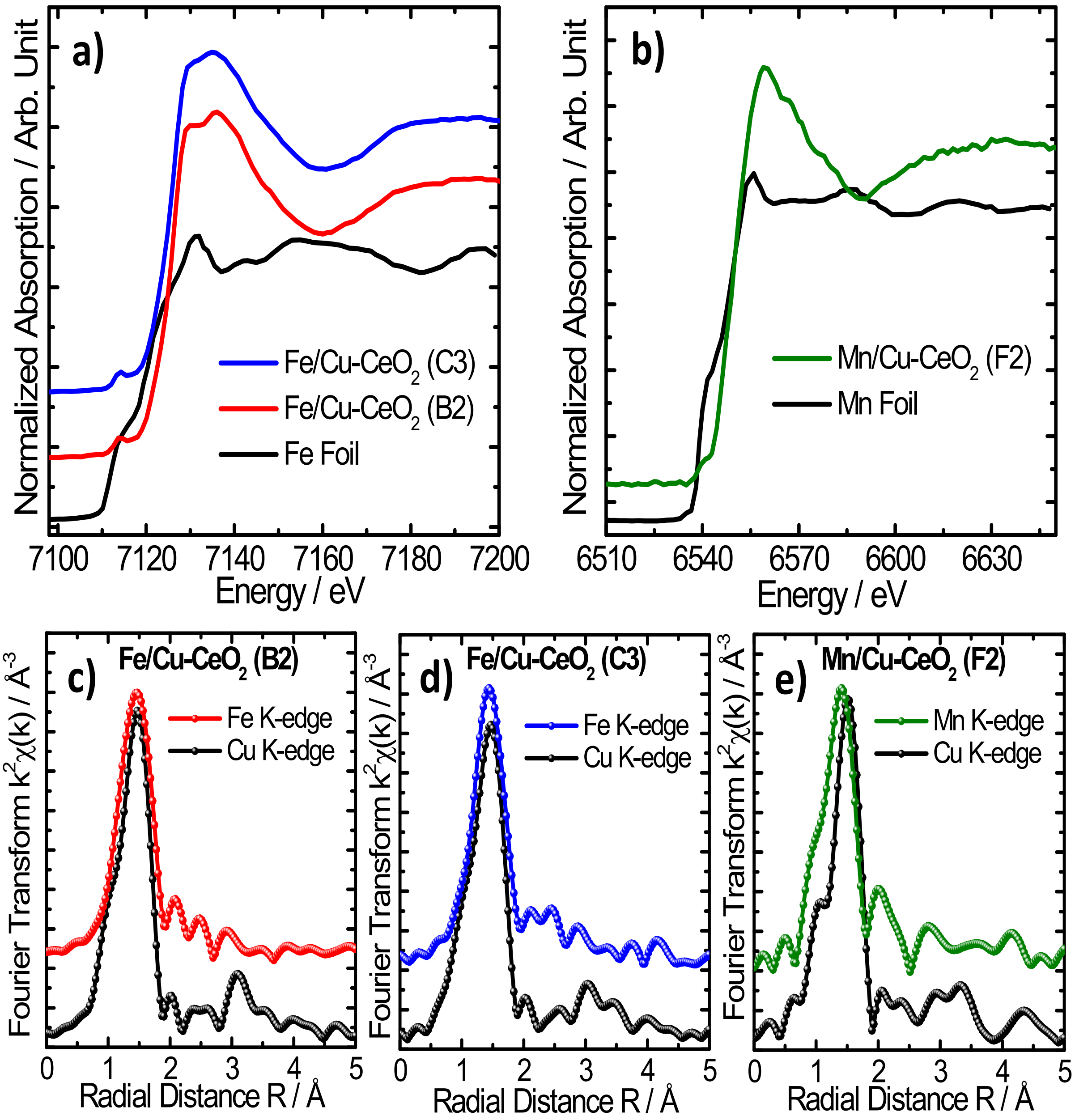

Figure S1: Normalized XANES spectra of the Fe/Cu-CeO$_2$ (B2, C3) (a) and Mn/Cu-CeO$_2$ (b) measured at the Fe K-edge (7112 eV) and Mn K-edge (6539 eV), respectively. Fourier transforms of the *χ(k)* at Cu K-edge (8979 eV) and Fe K-edge (7112 eV) for the Fe/Cu-CeO$_2$ (B2) (c) and Fe/Cu-CeO$_2$ (C3) (d), and Mn K-edge (6539 eV) for the Mn/Cu-CeO$_2$, (F2) (e) nano-catalysts

1. Experimental set up for XAFS studies

The X-ray absorption fine structure (XAFS) measurements were performed on the XAFS/XRF beamline, Synchrotron-Light for Experimental Science and Applications in the Middle East (SESAME), operating at 2.5 GeV in “decay” mode with a maximum electron current of 250 mA. The Ce L_3-edge XAFS spectra of all the samples (CeO_2 host matrices) were measured in transmission mode at room temperature, monitoring X-ray beam intensity before and after the sample by two ionization chambers filled with a mixture of noble gases. Whereas, XAFS spectra of the *d*-transition metal ions (Cu, Fe and Mn) dopants were acquired in fluorescence mode with a Silicon Drift Detectors (SDD), KETEK GmbH, in their respective spectral range of Cu K-edge (8979 eV), Fe K-edge (7112 eV) and Mn K-edge (6539 eV), respectively, for all the samples. XAFS data were measured with a double-crystal Si (111) monochromator and energy was calibrated according to the corresponding absorption *K*-edge of Cu, Fe and Mn metallic foils. The samples were prepared in pallet form by pressing a homogeneous mixture of calculated quantity of finely ground material and polyvinylpyrrolidone (PVP) powder. The amount of material in each pellet was calculated using XAFS mass software to give an absorption μ_t~1.5, just above the Zn absorption K-edge.

1.1 Experimental set up for EXAFS analysis

In order to investigate the local chemical environment around the Ce ions, the Ce L_3-edge extended X-ray absorption fine structure (EXAFS) spectra of all the samples were quantitatively analyzed and nonlinear best fit was performed to their Fourier transformed k^3-weighted experimental EXAFS signals, using Artemis program from the Demeter. The EXAFS data were preprocessed in Athena from Demeter, including background subtraction, spectrum alignment and normalization, whereas the background R_{bkg} was set to 1.1 Å. The theoretical values of effective scattering amplitude $F_i(k)$, effective scattering phase shift $\Phi_i(k)$ and mean free path of the photoelectron λ were calculated from the CeO_2 cubic structure of lattice parameters (a, b and c = 5.411 Å) and F m -3 m (225) space group *via* FEFF8-lite code in Artemis, generating various scattering paths. The structural EXAFS parameters were determined from the best fit to data, employing IFEFFIT implemented in Artemis. These structural parameters include degeneracy of the path (N_{degen} or N), change in the half-path length (ΔR), mean square relative displacement about the equilibrium path length (σ^2), passive electrons reduction factor (S_0^2) and energy shift of the photoelectron (ΔE_0).

In Artemis fitting procedure, the goodness of the fit is usually evaluated by effective minimization of the value of standard χ^2 fitting metric that can be described by Eq. 1:

$$\chi^2 = \frac{N_{idp}}{\varepsilon N_{data}} \sum_{i=min}^{max} [Re(\chi_d(r_i) - \chi_t(r_i))^2 + Im(\chi_d(r_i) - \chi_t(r_i))^2] \tag{1}$$

where $\chi_d(r_i)$ and $\chi_t(r_i)$ represent the real (Re) and imaginary (Im) components of the Fourier transform of experimental EXAFS data and theoretical $\chi(k)$, respectively, and ε is the estimated uncertainty in the measured data. It is obvious from Equation 1 that the χ^2 is normalized by the uncertainty in the measurement (ε). Therefore, it is quite common that the value of the reduced χ^2 is observed much larger in the fit, because the estimate of the uncertainty in the measurement (ε) is not always reliable. The reduced χ^2, also represented as χ^2_v can be obtained by dividing the χ^2 with number of degrees of freedom in the fit (v). The value of v is derived from subtraction of the number of actual variables or parameters in the fit from the number of independent points (N_{idp}) in the fit[6], whereas N_{idp} can be determined by Eq. 2:

$$N_{idp} \approx 2\Delta k \Delta R / \pi \tag{2}$$

where Δk and ΔR are the range of the EXAFS data in k- and R-space under fit analysis, respectively. Accordingly, the goodness of the fit was determined by minimizing an alternate statistical parameter, EXAFS reliability factor R. The R_{factor} is the sum of the squares of the differences of real and imaginary parts of the Fourier transform of the data and theory divided by the sum of the squares of the data, as described earlier in the literature.

The best fits to the Ce L_3-edge EXAFS data were performed in R-space from 1.1 to 2.3 Å interval with Hanning window and in the 2-10 Å^{-1} k range. The only shell of the Ce–O single scattering path was included in the best fit analysis due to the small energy separation between L_3 and L_2 edges. The amplitude reduction factor (S^2_0), energy shift parameter (ΔE_0) and disorder in the bond length or mean-square relative displacement (MSRD), also known as Debye-Waller factors (σ^2) was refined to get the best fit result.

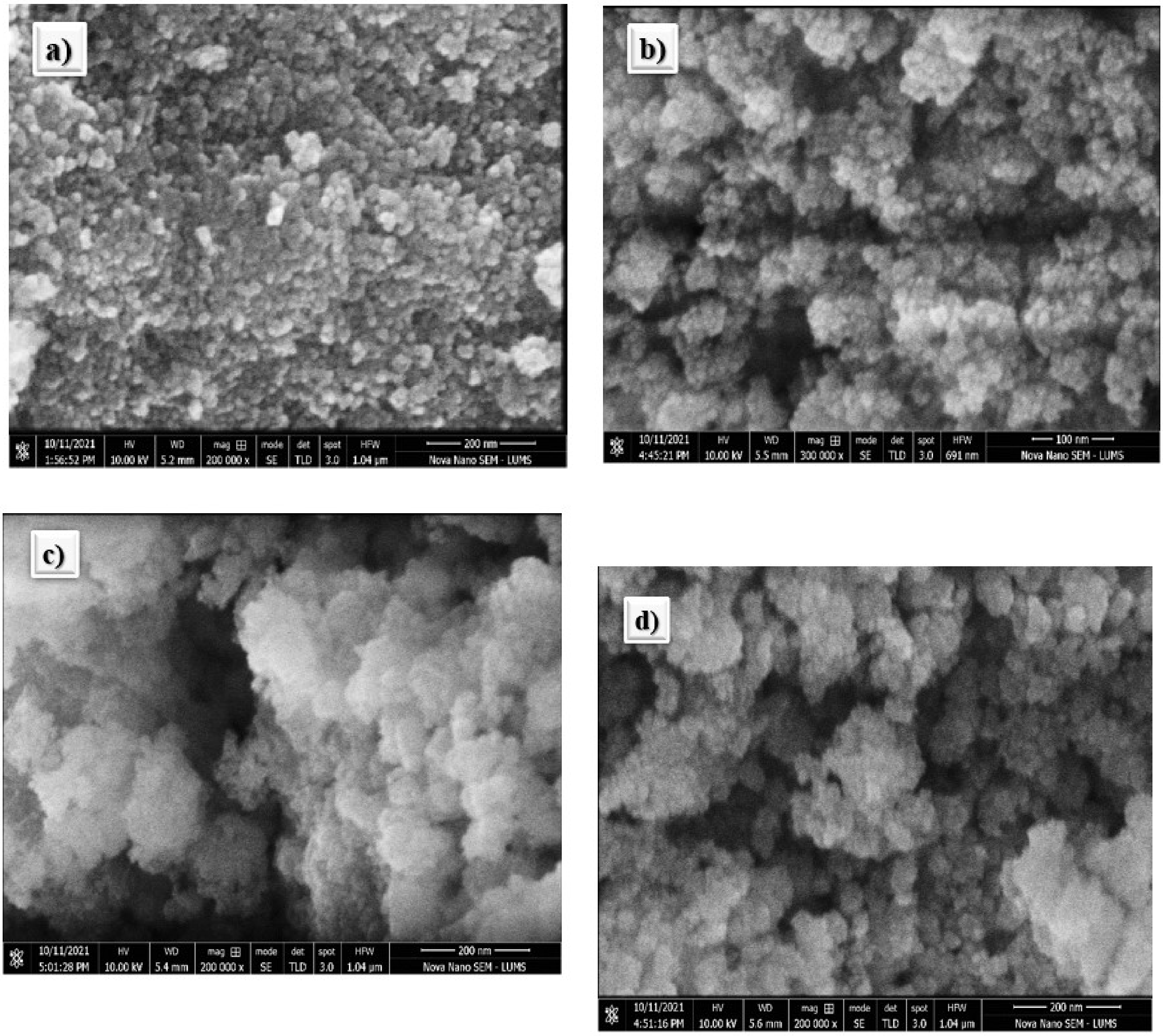

Figure S2: SEM images of a) Mn/Fe-CeO_2, b) Cu/Ag-CeO_2, c) Fe/Cu-CeO_2 and d) Mn/Cu-CeO_2

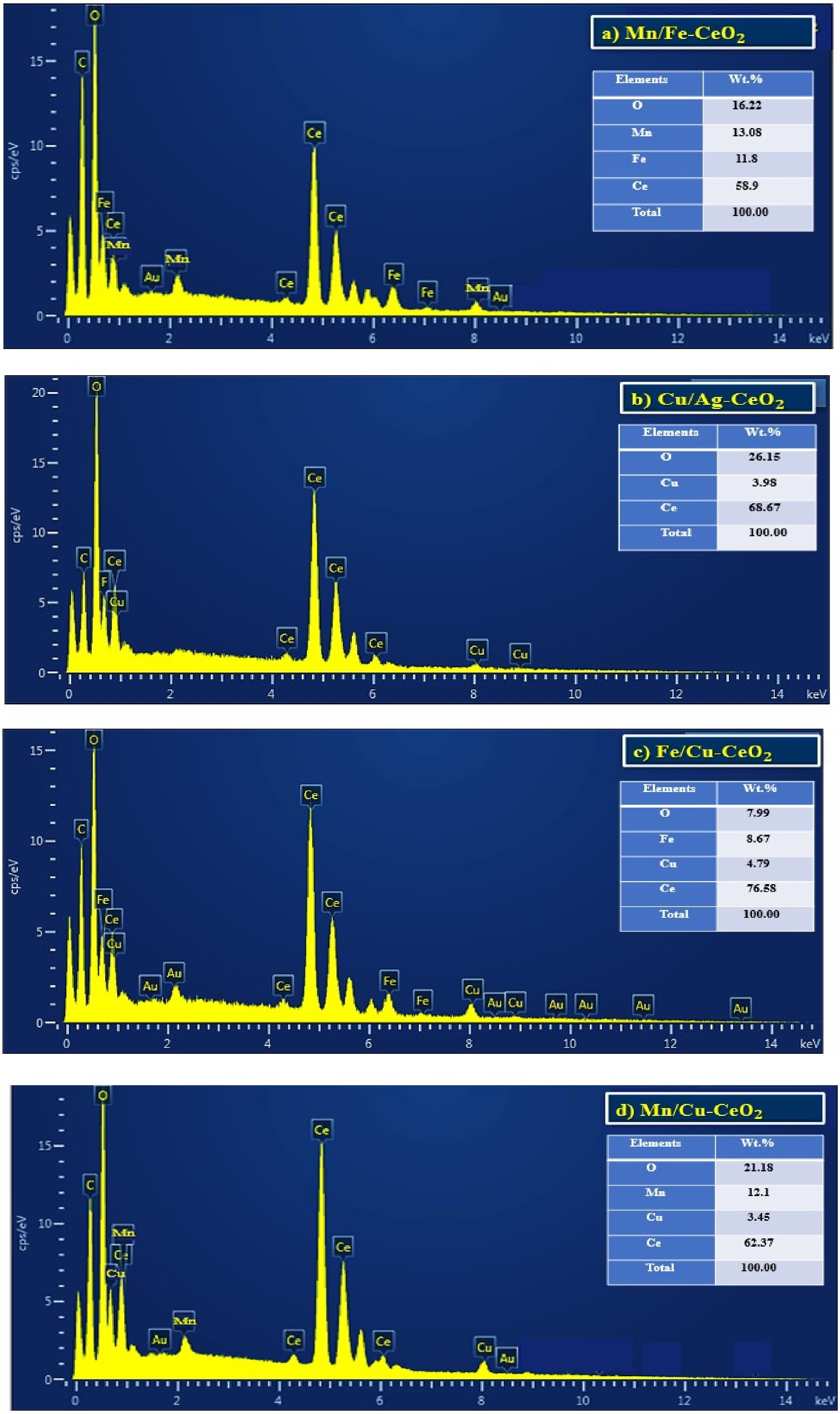

Figure S3: EDX results of transition metal co-doped ceria nano-additives

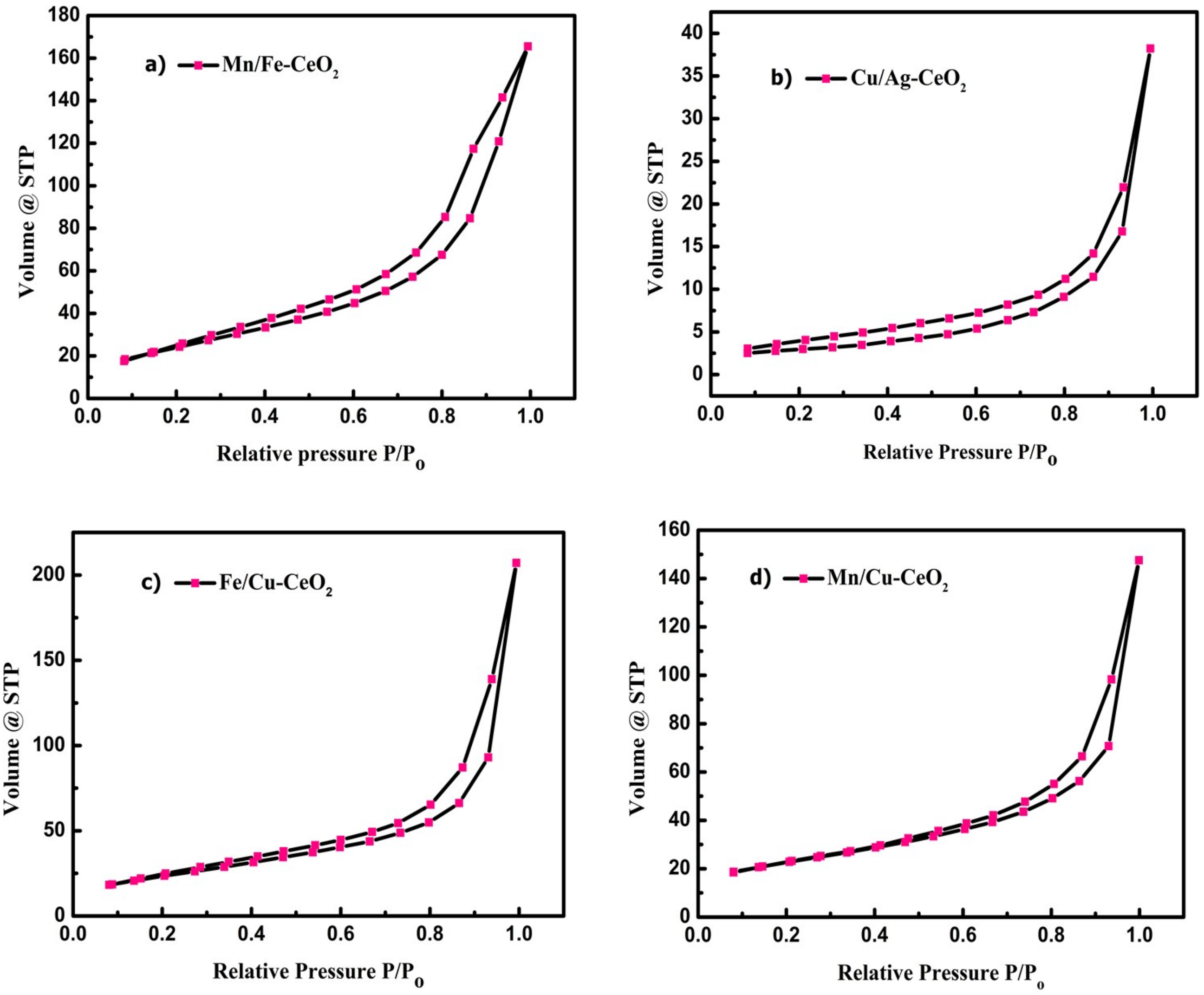

Figure S4: N_2 adsorption isotherm of cerium oxide-based nano-catalysts
